# Applying the emergency risk management process to tackle the crisis of antibiotic resistance

**DOI:** 10.3389/fmicb.2015.00927

**Published:** 2015-09-04

**Authors:** Dale Dominey-Howes, Beata Bajorek, Carolyn A. Michael, Brittany Betteridge, Jonathan Iredell, Maurizio Labbate

**Affiliations:** ^1^Asia – Pacific Natural Hazards and Disaster Risk Research Group, The School of Geosciences, The University of SydneySydney, NSW, Australia; ^2^The UTS Graduate School of Health, University of Technology SydneySydney, NSW, Australia; ^3^The School of Life Sciences, University of Technology SydneySydney, NSW, Australia; ^4^Westmead Millennium Institute for Medical Research and The Marie Bashir Institute, University of SydneySydney, NSW, Australia; ^5^The ithree Institute, University of Technology SydneySydney, NSW, Australia

**Keywords:** antibiotic resistance, disaster risk, risk perception, emergency management

## Abstract

We advocate that antibiotic resistance be reframed as a disaster risk management problem. Antibiotic-resistant infections represent a risk to life as significant as other commonly occurring natural disasters (e.g., earthquakes). Despite efforts by global health authorities, antibiotic resistance continues to escalate. Therefore, new approaches and expertise are needed to manage the issue. In this perspective we: (1) make a call for the emergency management community to recognize the antibiotic resistance risk and join in addressing this problem; (2) suggest using the risk management process to help tackle antibiotic resistance; (3) show why this approach has value and why it is different to existing approaches; and (4) identify public perception of antibiotic resistance as an important issue that warrants exploration.

## The disaster of antibiotic resistance

On the 1st May 2014 the World Health Organization (WHO) (World Health Organization, 2014) in its first global assessment of antimicrobial resistance reported that antibiotic resistance has reached alarming proportions. It is no longer a future problem but a contemporary reality. This statement reinforces decades of calls for action from scientists, clinicians, and leading authorities as well as contemporary authorities such as the Deputy Director of the Center for Disease Control (CDC) who stated we have reached the “end of the antibiotic-era” and the UK Chief Medical Officer, Professor Dame Sally Davies who called for antibiotic resistance to be placed on the UK National Disaster Risk Register ahead of terrorism. Significantly, following Professor Dame Sally's call, antimicrobial resistance was listed on the UK National Risk Register of Civil Emergencies. The Register estimates that up to 80,000 people could die from a single antimicrobial-resistant outbreak (UK Cabinet Office, 2015). The tone of the language used by these individuals and organizations to describe the crisis of antibiotic resistance is frightening. It is also reminiscent of that used by emergency managers to describe the unfolding processes of drought and global climate change and commonly occurring disasters such as earthquakes, floods and storms.

A disaster is defined as “a serious disruption of the functioning of a community or a society, involving widespread human, material, economic or environmental losses and impacts, which exceeds the ability of the affected community or society to cope using its own resources” (United Nations Office for Disaster Risk Reduction, 2009). Antibiotic-resistant infections are rising fast and affect millions of people globally, disrupting the functioning of societies and outpacing the coping capacity of existing health and medical communities. Consequently, we contend that antibiotic resistance has become a slow-onset disaster that, like climate change, has struggled to elicit the sort of coordinated international response that is required to deal with a crisis of this scale (Woolhouse and Farrar, 2014). Anthropogenic modification of the Earth's climate system leading to slow-onset climate change provides the foundation for sudden-onset natural disasters such as hurricanes and storms (Intergovernmental Panel on Climate Change, 2012). Likewise, increasingly widespread slow-onset antibiotic resistance lays the foundation for the future occurrence of sudden-onset bacterial epidemic and pandemic disasters. Significantly, antibiotic resistance has not been recognized by emergency management authorities as a major risk to life generally, or as a factor that will contribute to the severity of future bacterial epidemics and pandemics. This is despite the fact that antibiotics are one of the key tools used to respond to epidemic and pandemic outbreaks (World Health Organization: Regional office for the Western Pacific, 2011). However, sudden-onset antibiotic-resistant infectious outbreaks do already occur in hospitals, nursing homes and other settings affecting the most vulnerable and offering insights into what a future antibiotic-resistant epidemic or pandemic outbreak might look like. These outbreaks require costly interventions. For example, in the United States, more than 2 million people are affected by antibiotic-resistant infections per year with at least 23,000 annual deaths. Notwithstanding the extraordinary human loss, this equals US$20 billion in extra healthcare costs and US$35 billion in lost productivity (Center for Disease Control, 2013). In Australia, it is estimated that antimicrobial resistance costs the health care budget AU$250 million per annum and costs the community as much as AU$500 million per year (Shaban et al., 2013). Interestingly, when focusing just on loss of human life associated with high profile natural disaster events in different parts of the world that resulted in significant risk management policy and practice changes, antibiotic-resistant infections are taking many more lives. For example, Hurricane Katrina in 2005 in the USA took over 1800 lives and the Black Saturday Bushfires in Australia took 178 lives.

Humanity has become highly dependent on antibiotics. They are utilized to help people combat bacterial infections, to prevent bacterial infection in high-risk individuals such as those undergoing invasive surgical procedures, to increase food output in agriculture and to prevent infectious outbreaks following natural disasters. In the past, the emergence of antibiotic resistance was circumvented by the development of new antibiotics that helped maintain our coping capacity. As such, resistance was not really an issue. Unfortunately, drug discovery and development is expensive and it is estimated that ~US$1 billion is spent before a drug even reaches the market, hence this approach has stalled due to various economic and regulatory constraints (Power, 2006). Due to the rate at which bacteria can become resistant, a pharmaceutical company may only achieve as little as 1–2 years revenue before drug effectiveness in clinical medicine begins to diminish (Power, 2006). Furthermore, courses of treatment are short, typically 1 week, so the capacity to generate profit is modest. Consequently, humanity faces a situation where the existing antibiotics are losing their effectiveness and there is a dearth of new antibiotics coming onto the market. Although research into new drugs (Ling et al., 2015), alternative therapies such as honey for treating wounds (Lu et al., 2013), herbal remedies (Tiwari et al., 2015), and nanomaterials (Tiwari et al., 2014) all show promise, it appears that society's current ability to cope with the problem has reached its limit and further resources are needed.

Much has been said about the reasons for the evolution of antibiotic resistance, which is highly complex and multidimensional. However, in its most basic form, antibiotic use leads to antibiotic resistance (for a review on the evolution of antibiotic resistance see Michael et al., 2014). Widespread use, overuse and misuse of antibiotics in multiple settings, including human medicine and agriculture, have created strong selection pressures in favor of those bacteria that resist antibiotics. Consequently, resistant bacteria are increasingly found directly on or in humans and our companion animals, farm animals, seafood, fruit, vegetables and in the natural environment (Baquero et al., 2008; Kemper, 2008; Wright, 2010; Nesme et al., 2014). Apart from developing resistance via genetic mutation (Maharjan et al., 2006; Beardmore et al., 2011), spread of resistance to pathogenic bacteria can also occur via a combination of two processes. Firstly, bacteria efficiently acquire resistance genes from other bacteria i.e., lateral gene transfer (Labbate et al., 2009). Secondly, through the consumption of food or direct contact with people or environments containing antibiotic-resistant bacteria, normal healthy bacteria in and on humans acquire resistance which can then pass on to disease-causing bacteria during an infection (van Hal et al., 2009).

To date, responsibility for responding to the risk associated with antibiotic resistance has fallen to the health and medical industries. Globally, these experts have done a tremendous job in attempting to manage this issue. Given the scale of the crisis, extensive resources have been invested into “top-down” antimicrobial stewardship (AMS) programs, thereby addressing this issue from a “service” model perspective more akin to disease management or quality improvement processes (Center for Disease Control, 2014). The range of the activities falling under this AMS umbrella are diverse, although mostly implemented within hospital settings in higher-income countries, and have been evaluated using various outcome and process measures (Looke and Duguid, 2011; Hahn and Doby, 2013). Despite this, it is recognized that antimicrobial resistance persists, as does the over-utilization of antimicrobials (Laxminarayan et al., 2013). Therefore, looking at the problem from different perspectives may be of value. Critically, for the health and medical industries, dealing with antibiotic resistance is surpassing their capacity to contain and manage the problem. The distinctive shift in the tone of the language used by the WHO to that of disaster risk management presents an opportunity to take just such a new perspective. Consequently, we propose rebranding antibiotic resistance as a disaster risk management problem and argue that the methods used to support communities in preparation for disasters may be applied to supporting communities prepare for the risk associated with increasing levels of antibiotic resistance.

Since the slow-onset emergence of antibiotic resistance forms the foundation and trigger for an inevitable sudden-onset epidemic or pandemic disaster, disaster risk management provides a novel way of thinking about responding to the risk now. It provides a useful process and set of tools to engage with the general public and other stakeholders in order to prepare for and manage the risk of antibiotic-resistant bacterial infections generally, and bacterial epidemics and pandemics specifically. This is because the public is, in a sense, already primed to the value of disaster risk management. By analogy, if thousands of people were dying each year in slow-onset disasters like droughts or rapid-onset disasters like bushfires, floods or hurricanes, this would be considered a very serious “disaster risk management problem.” Bacteria are biological risks to life and as such they fit within the domain of disaster risk management. Given that tens of thousands of people are dying each year due to the acquisition of antibiotic-resistant bacterial infections (European Centre for Disease Prevention and Control/European Medicines Agency, 2009; Center for Disease Control, 2013), the implications for the field of disaster risk management become obvious. That is: (1) antibiotic and antimicrobial resistance is a risk to life; (2) fatalities are already unacceptably high; (3) the placement of antibiotic resistance on the national risk register triggers the need for an emergency management response; and (4) emergency managers have the skills, tools and experience to engage with communities to reduce the risk (e.g., via supporting positive behavioral change).

## The emergency risk management process

Disasters take lives, impact individuals, families and communities, cause devastation and disrupt our socio-economic and ecological systems (Adger et al., 2005). Around the world, legislation directs emergency management authorities at national and local levels to develop “disaster risk registers” that identify risks that threaten society. Subsequently, those authorities must then develop emergency management and response plans for each identified risk to prepare for, and respond to, emergencies and disasters using the “Emergency Risk Management Process.” Sudden-onset bacterial epidemics and pandemics already fall under the Emergency Risk Management Process (ERMP) because they are biological risks to life. Here we call for antibiotic resistance to be placed on national risk registers everywhere as has been done in the UK. This would automatically raise the profile of the problem and force response plans using the ERMP.

It makes sense that emergency management participate in addressing this problem as antibiotic resistance already affects their capacity to manage disasters. For example, one of the major tools used by Health Emergency Managers (e.g., the NSW State Health Emergency Management Unit in Australia) to manage the risk associated with bacterial epidemics and pandemics is antibiotics (World Health Organization: Regional office for the Western Pacific, 2011). The emergence of antibiotic resistance means that the capacity of government authorities to manage the risk is seriously compromised, particularly in poorly resourced settings where access to services is limited and where reductions in mortality have historically been dependent on antibiotics alone (Okeke et al., 2005). This also includes bacterial infectious epidemics that come off the back of a primary disaster such as an earthquake (e.g., the 2010 Haiti earthquake) or another infectious outbreak such as viral influenza leading to secondary bacterial pneumonial infections. In fact, secondary bacterial pneumonial infections have been suggested as the main reason for the 50–100 million deaths during the 1918 spanish flu pandemic (Brundage and Shanks, 2008).

**Figure 1:**
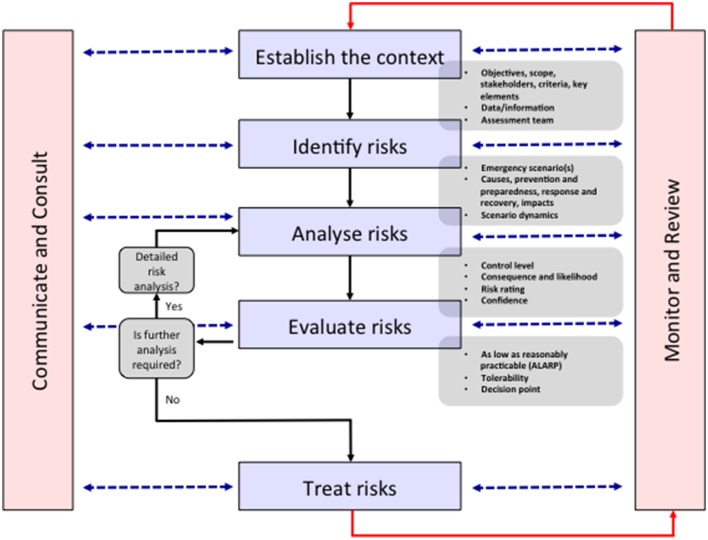
**The emergency risk management process**. In the context of reframing antibiotic resistance as a disaster risk management problem, all stakeholders are identified in the “Establish the context” stage. An indicative list of stakeholders is shown in Table 1. The identifying, analyzing, evaluating and treating risk stages are undertaken via communication and consultation with stakeholders and monitoring and reviewing by the assessment team. This “bottom-up” approach leads to more successful policy outcomes by establishing solutions consistent with the socio-cultural context of the stakeholders. Modified from The Australian Government (2010).

The ERMP is described as being integral to management and decision-making, integrated into practices and culture and tailored to a specific community's risk profile. It is a systematic method for identifying, analyzing, evaluating and treating risks and takes an iterative approach with well-defined activities that lead to implementation of risk-treatment strategies (Figure 1). The process entails (1) establishing the context, (2) identifying the risks, (3) analyzing the risks, (4) evaluating the risks, and (5) treating the risks. This 5-step process is supported by two enabling activities—communicating and consulting, and monitoring and reviewing (The Australian Government, 2010). “Risk Assessment” comprises the identification, analysis and evaluation of the risk components of the ERMP, and must be undertaken alongside “communication and consultation” with all stakeholders. Indeed, the antibiotic resistance problem is made up of multiple stakeholders (Table 1), existing in a complex network that is not fully understood, but who must all be consulted to ensure that any inroads made in one area are not circumvented through alternative actions. For example, patients prevented from acquiring antibiotics by improved AMS programs may self-source them from internet pharmacies or hoard antibiotics acquired through prescription (Michael et al., 2014). To show the value of the ERMP, the following section will focus on the general public as a specific stakeholder.

**Table 1:** **Representative list of stakeholders and their potential role(s) in antibiotic-resistance**.

**Stakeholder**	**Role(s)**
Public/Community	User of antibiotics for human health; At risk from antibiotic-resistant infections; Demand for cheap meat from food industry
Prescribers	Prescriber of antibiotics to patients/public
Pharmacists	Supply of antibiotics; Educating the public regarding proper antibiotic; Drug utilization evaluation; Medication review and therapeutic adjustment
Food industry (Agriculture and distribution)	Use of antibiotics in agriculture; Demand by distributors to provide cheap meat
Wastewater management	Treatment of wastewater containing antibiotics; Non-removal of antibiotics from wastewater allows entry into the environment facilitating resistance
Pharmaceutical companies	Manufacture of antibiotics; Drug discovery; Marketing of antibiotic use to various stakeholders
NGOs/Community groups/Lobby groups	Education, influencing or lobbying various stakeholders (e.g., anti-vaccination lobby or National Prescribing Service)
Government	Policy development and regulation and control
Scientific researchers	Research into new drugs, vaccines etc.; Evidence for driving policy and education
Research funding bodies	Funding research for antibiotic resistance
Media	Mass education and influence of public

## How is the ERMP different from other risk management strategies used in addressing antibiotic resistance?

The most recent WHO and CDC reports (Center for Disease Control, 2013; World Health Organization, 2014) on antimicrobial resistance outlined a series of action items indicating that a “top-down” approach to risk management had been utilized—that is, that solutions “for the community” are largely being directed from above rather than being “developed in consultation” (i.e., from within) with target stakeholder groups or communities. For example, AMS programs are a top-down approach to managing antibiotic resistance to which extensive resources have been allocated. The ERMP is a “bottom-up” approach that identifies solutions to a risk problem tailored to a specific community in consultation with that community. This is an important point of difference because solutions developed for one community are not necessarily appropriate to translate to another. Also, “outsiders” that do not understand how a stakeholder group functions, or the socio-cultural and behavioral practices that establish the context for community behavior and decision making may impose, all be it well meaning, regulations and practices, that are completely intolerable to the target stakeholder group.

For example, northern Australia is regularly affected by tropical cyclones. Small remote communities comprising dominantly of Indigenous and Torres Strait Islander people are spread out over wide areas. In recent years as tropical cyclones have approached and emergency management authorities made the decision to evacuate people from these remote communities by plane, actual emergency management officials on the ground found themselves having to account for significant cultural differences between the white Australian population and the Indigenous peoples living in those places. Specifically, Indigenous communities have complex governance structures and cultural relations within and between kinship groups. The initially “top-down” approach of emergency management simply saw everyone as the same and wanted (intensions were well meaning here) to get everybody on to the aircraft and safely away as quickly as possible. However, for the Indigenous people, complex socio-cultural relationships meant that it was not possible for “this person” and “that person” to be sat next to each other openly in the small space of an aircraft interior. This would “poison” relationships within the community that would have long lasting and devastating impacts. Consequently, the Indigenous people refused to comply with evacuation orders. As a result, a “bottom-up,” negotiated arrangement was developed that satisfied the complex socio-cultural needs of the target stakeholder community. The answer was as simple as hanging a sheet inside the aircraft that divided space into safe and respected compartments (Veland et al., 2010).

Examples like the one just described show how when policies and procedures are negotiated from the bottom-up they are owned by the communities to which they are targeted. More often than not, they are successful. The reverse is also the case and policy failure frequently occurs due to poor community consultation. If we consider that antibiotic resistance is a slow-onset disaster then to prevent future policy failures around antibiotic prescription and use, consultation with diverse communities and tailoring solutions specific to those communities will be crucial. For example, education programs about antibiotic use may need to be tailored to specific communities socio-cultural and behavioral contexts, and in languages other than English, since the factors driving antibiotic demand are likely to be diverse.

Another key defining element of the ERMP is that emphasis is placed on empowering individuals, families, communities or any other stakeholder, to take responsibility for the risks they face. In this context, once again, the emergency management approach offers a novel perspective where rather than having governments and health professionals take full responsibility, the general public will need to take personal responsibility for the risk they face from antibiotic resistance—once of course they understand that risk and their own role in enabling, or limiting that risk. Microbiologists understand that selection pressure from antibiotic overuse results in increased resistance and that minimizing use is crucial to slowing down the rate of resistance spread. However, how to implement this change in stakeholders is the challenge. Antibiotic resistance is not just a microbial problem—it is also a socio-cultural and behavioral problem. In the context of the issue of the emergence of antibiotic resistance and its effect on the risk profile of epidemics and pandemics, poor stakeholder risk perception underpins inappropriate behaviors, such as patients pressuring clinicians by demanding antibiotics for viral infections, or patients' non-adherence to courses of antibiotics (Michael et al., 2014). Importantly, lacking is a comprehensive understanding of how different stakeholders perceive antibiotics, particularly the problem of antibiotic resistance (Radyowijati and Haak, 2003; Norris et al., 2013; Hu and Wang, 2015). A thorough understanding of these factors is necessary to guide the development of effective stakeholder education programs and strategies, and thereby reduce risk and increase resilience to the risk of antibiotic resistance and resistant infections. Apart from preserving current antibiotics, modifying behavior will help preserve future drugs for which substantial resources have been and will be invested. Consequently, missing from the ERMP is crucial data to assess the risk and to synthesize strategies to manage the risk. The implications for governments and their agencies for managing the disaster risk of epidemics and pandemics is problematic.

The academic and practitioner fields of disaster risk management have developed an extensive body of literature on how poor or low stakeholder perceptions of risks and disasters led to inapproriate and poor behavior (Bird, 2009; Hoppner et al., 2012). Determining why stakeholders have the risk perceptions they do is not difficult to investigate. In fact, since the middle of the 20th century an entire field of research has evolved to explore risk perception and risk behavior that has fractured in to several distinct schools of thought (e.g., the psychometric, the socio-cultural, and the social amplification of risk schools). Risk managers, together with hazard perception experts, play a critical role here. Experienced in investigating why communities behave the way they do, and hold the views and perceptions of risks that they do, and in communicating complex risk information to different stakeholders, risk management can help (Wolf and Moser, 2011; van der Linden, 2015). A variety of theoretical models are available that explore cognitive, psychological, behavioral and socio-cultural factors that influence risk perception and behavioral adjustment and these have been robustly tested in multiple hazard contexts. For an excellent summary of these, see the seminal work of Paul Slovic and the references therein (Slovic, 2000).

In light of this, we propose shifting the discourse to one of increased multiple stakeholder engagement and awareness, thereby rebranding of the issue of antibiotic resistance away from one exclusively of “general health, medicine, and molecular microbiology” to one of “disaster risk reduction” (Dominey-Howes et al., 2014). It is our perspective that the slow-onset emergence of antibiotic resistance is, in fact, a risk that ought to be framed as a disaster risk management problem—much like that of slow-onset climate change. Consequently, we call for the emergency management community, and socio-behavioral experts in risk perception, to recognize the threat that the emergence of antibiotic resistance presents both generally, and to treating the risk of bacterial epidemics and pandemics specifically. Further, we seek a cooperative partnership with the medical and health and molecular microbiology communities to address this problem—traditionally within their domain—as a wider interdisciplinary research and policy problem. To that end, a number of initial actions might include (1) establishing an International Panel on Antimicrobial Resistance similar to the Intergovernmental Panel on Climate Change (which was recently raised by Woolhouse and Farrar (2014); (2) more international, interdisciplinary conferences and workshops that bring together health and medical experts with microbiologists and emergency managers and policy makers to explore common ground and build mutual understanding and awareness and (3) traditional medical and microbiological conferences and workshops focusing on antibiotic resistance hosting special sessions dealing exclusively with community knowledge and engagement, risk management and policy in order to broaden the perspectives examined. We believe this will both appropriately raise the profile of antibiotic resistance in the wider community and provide new ideas and innovations for tackling the risk.

## Conflict of interest statement

The authors declare that the research was conducted in the absence of any commercial or financial relationships that could be construed as a potential conflict of interest.
